# Vertical movement symmetry of the withers in horses with induced forelimb and hindlimb lameness at trot

**DOI:** 10.1111/evj.12844

**Published:** 2018-05-17

**Authors:** M. Rhodin, E. Persson‐Sjodin, A. Egenvall, F. M. Serra Bragança, T. Pfau, L. Roepstorff, M. A. Weishaupt, M. H. Thomsen, P. R. van Weeren, E. Hernlund

**Affiliations:** ^1^ Department of Anatomy, Physiology and Biochemistry Swedish University of Agricultural Sciences Uppsala Sweden; ^2^ Department of Clinical Sciences Swedish University of Agricultural Sciences Uppsala Sweden; ^3^ Department of Equine Sciences Faculty of Veterinary Medicine Utrecht University Utrecht The Netherlands; ^4^ Department of Clinical Science and Services The Royal Veterinary College University of London North Mymms, Hatfield Hertfordshire UK; ^5^ Department, Vetsuisse Faculty University of Zurich Zurich Switzerland; ^6^ Department of Veterinary Clinical Sciences Faculty of Health and Medical Sciences University of Copenhagen Taastrup Denmark

**Keywords:** horse, compensatory lameness, withers asymmetry, gait analysis, objective lameness measurement

## Abstract

**Background:**

The main criteria for lameness assessment in horses are head movement for forelimb lameness and pelvic movement for hindlimb lameness. However, compensatory head nod in horses with primary hindlimb lameness is a well‐known phenomenon. This compensatory head nod movement can be easily misinterpreted as a sign of primary ipsilateral forelimb lameness. Therefore, discriminating compensatory asymmetries from primary directly pain‐related movement asymmetries is a prerequisite for successful lameness assessment.

**Objectives:**

To investigate the association between head, withers and pelvis movement asymmetry in horses with induced forelimb and hindlimb lameness.

**Study design:**

Experimental study.

**Methods:**

In 10 clinically sound Warmblood riding horses, forelimb and hindlimb lameness were induced using a sole pressure model. The horses were then trotted on a treadmill. Three‐dimensional optical motion capture was used to collect kinematic data from reflective markers attached to the poll, withers and tubera sacrale. The magnitude and side (left or right) of the following symmetry parameters, vertical difference in minimum position, maximum position and range‐up were calculated for head, withers, and pelvis. Mixed models were used to analyse data from induced forelimb and hindlimb lameness.

**Results:**

For each mm increase in pelvic asymmetry in response to hindlimb lameness induction, withers movement asymmetry increased by 0.35–0.55 mm, but towards the contralateral side. In induced forelimb lameness, for each mm increase in head movement asymmetry, withers movement asymmetry increased by 0.05–0.10 mm, in agreement with the head movement asymmetry direction, both indicating lameness in the induced forelimb.

**Main limitations:**

Results must be confirmed in clinically lame horses trotting overground.

**Conclusions:**

The vertical asymmetry pattern of the withers discriminated a head nod associated with true forelimb lameness from the compensatory head movement asymmetry caused by primary hindlimb lameness. Measuring movement symmetry of the withers may, thus, aid in determining primary lameness location.

## Introduction

Subjective visual evaluation and semi‐quantitative assessment of lameness is standard practice and aims to identify the affected limb(s) and localise the cause of pain or dysfunction. Scoring of mild to moderate lameness has been shown to be only ‘moderately reliable’ 1 or ‘just within acceptable limits’ 2, 3. Also, expectation bias influences subjective evaluation 4. There is, therefore, a need for a more unbiased and objective evaluation of lameness in the clinical decision‐making process. The main criteria for both visual and objective lameness assessment are asymmetries in the head movement for forelimb lameness and asymmetries in the pelvic movement for hindlimb lameness 5, 6, 7. However, compensatory movement asymmetry of the head, caused by primary hindlimb lameness, and compensatory movement asymmetry of the pelvis, caused by primary forelimb lameness, can be present during straight line trot 5, 6, 8, 9, 10 and lungeing 11. Compensatory head nod in horses with primary hindlimb lameness is the most prominent. This compensatory head movement asymmetry can easily be misinterpreted as a primary lameness of the ipsilateral forelimb. Discriminating compensatory asymmetries from primary directly pain‐related movement asymmetries is therefore, a prerequisite for successful lameness assessment. Misinterpretation of such compensatory movements may be an important factor contributing to the low inter‐observer agreement in lameness evaluations performed by veterinarians 3, 12.

Buchner *et al*. 5 found that the movement symmetry of the withers was affected in horses with moderate induced forelimb and hindlimb lameness. When forelimb lameness was induced, the withers exhibited a smaller vertical displacement during lame diagonal stance compared with sound diagonal stance. Also, in horses with forelimb lameness, the total upward movement amplitude of the withers from the lame limb stance phase minimum to the swing phase maximum was smaller. After induction of hindlimb lameness, the movement of the withers was also affected, but to a smaller degree. After induction of moderate lameness, a decrease in the upward movement amplitude of the withers, following push‐off from the diagonal limb pair including the lame hindlimb, was detected 5. Buchner *et al*. 5 concluded that withers asymmetry indices were less prominent compared with head and pelvic indices and may not contribute to the detection of mild lameness if used for motion analysis. This is in agreement with the results of Peloso *et al*. 13 and Kubber *et al*. 14.

In horses with concurrent ipsilateral head and pelvic movement asymmetries, it may be difficult to determine the origin of the head movement asymmetry. According to the study by Buchner *et al*. 5, in horses with concurrent ipsilateral (same sided) head and pelvic movement asymmetries, withers movement asymmetry would be observed towards different directions depending on whether the forelimb or the hindlimb is the primary source of lameness. Therefore, withers movement may be useful to distinguish between a head nod of primary or compensatory nature.

In a study by Pfau *et al*. 15 vertical head, withers and pelvis movement asymmetries were quantified from inertial sensors in 163 Thoroughbreds with natural gait asymmetries during trot‐ups on hard ground. The results indicated that the relationship between head and withers asymmetry (i.e. same‐sided or opposite‐sided asymmetry) predicts the relationship between head and pelvic asymmetry in 69–77% of horses. The direction of head versus withers movement asymmetry identified most horses with ipsilateral and contralateral head and pelvic movement asymmetries. However, it remained unknown whether the horses presenting with ipsilateral asymmetries were hindlimb lame and whether those with contralateral asymmetries were forelimb lame since lameness was not localised to a specific limb.

The current study aimed to investigate the association between head, withers and pelvis movement asymmetry in horses with induced forelimb and hindlimb lameness and to evaluate whether movement symmetry of the withers can be used to discriminate a compensatory head nod in horses with hindlimb lameness from a head movement asymmetry due to primary forelimb lameness. It was hypothesised that, for forelimb lameness, the head and withers would show synchronised asymmetries (e.g. both indicating right forelimb), while for hindlimb lameness, the head and withers will show movement asymmetries of opposite directions (e.g. pelvis indicating left hindlimb, head indicating left forelimb, and withers indicating right forelimb).

## Materials and methods

### Horses

Ten horses were included in the study. They were all considered clinically sound when examined by an experienced Diplomate of the American College of Veterinary Sports Medicine and Rehabilitation (M.A.W.). All were Warmblood geldings with an age distribution of 5–21 years and mean height of the withers 169 ± 6.3 cm (range 161–180 cm). Horses were trained regularly and used in jumping and/or dressage competitions at amateur level. Spherical reflective markers with a diameter of 24 mm and 19 mm were attached with double‐sided adhesive tape to define anatomical landmarks of the horse. Markers were attached to the poll (head), over the highest point of the withers (withers), on the midline between the tubera sacrale (pelvis) and laterally to the left metatarsus.

### Lameness induction

Each horse was shod with modified horseshoes with a nut (thread M10) welded to the inner rim of each branch between the quarters and the bars, at the point of greatest width 16. Lameness was induced by screwing bolts with flat tips into the nuts of the modified horseshoe, thereby inducing pain by pressure on the corium of the sole. The procedure was controlled using a torque metre with 0.1 Nm increments (Type 757, Rahsol Dremotec^1^) to ensure that the same torque was applied to the medial and lateral side of the hoof. The goal was to induce three different degrees of reversible supporting lameness in each horse, evaluated subjectively by an experienced clinician (M.A.W.). The three degrees of lameness were defined as follows: 1 subtle lameness: irregularity not visible on every stride at the trot; 2 mild lameness: visible on every stride at the trot; and 3 moderate lameness: distinctly visible at the trot but without obvious disturbance to the cadence of movement.

### Data collection

Kinematic data were collected on a treadmill (Mustang 2200^2^ ) at trot using 10 infra‐red three‐dimensional optical motion capture cameras (Oqus 300+^3^ ) capturing at a frame rate of 256 Hz. Subjective evaluation of lameness grade was conducted during data collection. For some horses, more than three measurements were needed to achieve the three different lameness grades. This resulted in 1–7 measurements, with different torques for each limb induction in each horse. All trials were included in the analysis. In one horse, only a single induction was performed in one of the forelimbs due to an excessive lameness response. Fore‐ and hindlimb lameness was induced on separate days, and the order was randomised. Additional measurements without induction were carried out each day before induction and in between different limb inductions to verify return to baseline (data not shown).

### Data processing

The reconstruction of the three‐dimensional coordinates of each marker was automatically calculated by the Motion capture software Qualisys Track Manager^3^ (version 2.15). Each marker was identified and labelled using an automated (AIM model) and manual tracking. Raw data of the designated markers were exported to Matlab2017a^4^ for further analysis using custom‐written scripts. Stride segmentation was performed using the maximum protraction of the left hindlimb calculated from the metatarsal markers. The vertical displacement signal of head, withers and pelvis was high‐pass filtered using a 4th‐order zero‐phase Butterworth filter with the cut‐off frequency adjusted, based on the stride frequency of the horse in each trial. Different filters were evaluated (F. M. Serra Bragança, C. Roepstorff, T. Pfau, P. R. van Weeren M. Rhodin and L. Roepstorff, unpublished data) to find the most suitable method.

The inbuilt speedometer registered the speed of each trial in the treadmill, and stride duration was calculated as the time between consecutive strides from the stride‐segmented data from the pelvis marker.

**Figure 1:**
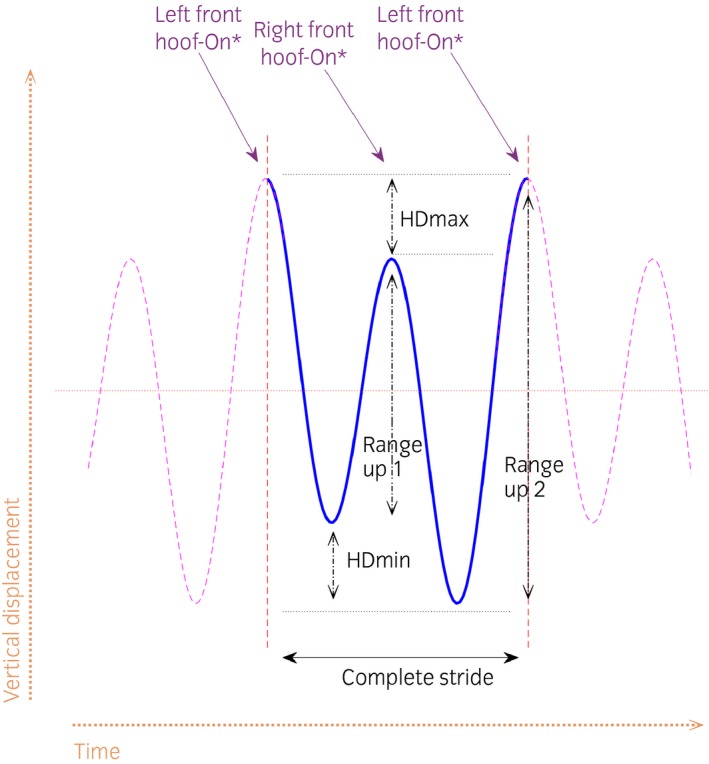
Example of vertical head movement in a horse with left forelimb lameness resulting in negative HDmin, HDmax and HDup (RangeUp1–RangeUp2) values. Pelvic and withers movement asymmetries are calculated in the same way but from vertical withers and pelvis movement signals.

For each stride, nine symmetry parameters were calculated using the vertical displacement of the head (poll), withers and pelvis markers (tubera sacrale). For each stride, the differences between the two displacement minima of the head (HDmin), pelvis (PDmin) and withers (WDmin), the difference between the two displacement maxima (HDmax, PDmax, WDmax), and the difference between vertical upward movement amplitude ‘range‐up’ (HDup, PDup, WDup) were calculated (Fig 1). For each trial, the mean value of all strides for each parameter was calculated. A horse presenting with reduced head or withers movement during left forelimb stance and push‐off is referred to as left forelimb asymmetric. A horse presenting with reduced pelvic movement during left hind stance and push‐off as left hind asymmetric 5. To include only successful inductions, forelimb induction trials with HDmin absolute mean values of ≥6 mm were selected. Likewise, hindlimb induction trials with absolute mean values of ≥3 mm for PDmin were selected. These thresholds for forelimb and hindlimb lameness are used in a commercially available lameness measurement system and agree with limits of repeatability of the system 17. In addition to studying effects across all horses, a subset of the hindlimb induction data was used to examine whether more pronounced compensatory head movement asymmetry would influence the movement pattern of the withers. For this, trials with successful hindlimb inductions showing a compensatory ipsilateral (same sign of PDmin and HDmin) head movement asymmetry (absolute mean values of ≥6 mm of HDmin) were selected.

### Data analysis

To exclude strides with excessive head movement, strides with HDmin, HDmax or HDup outliers outside the range of two SDs from the trial mean were automatically removed. The initial dataset consisted of 5842 strides, and after removal of outliers, 5365 strides remained (8% of strides removed as outliers).

To evaluate associations of head and pelvis variables with withers variables, stride‐level data were analysed with r studio (version 3.2.1^5^ ), using package nlme (version 3.1‐131) for mixed modelling. Head parameters were used as independent variables in the forelimb lameness analysis and pelvis parameters in the hindlimb lameness analysis, respectively. Dependent variables were investigated for a reasonable transformation close to normality, using normal probability plotting and examining for skewness and kurtosis. Random effects were horse and trial within horse. Head and pelvis variables, as well as stride duration, along with their squares and cubes, were initially tested in the models to examine the nonlinearity of these variables versus the modelled outcomes. Cubes were used because the distributions of these parameters are theoretically symmetrical around zero. Also, a third‐degree polynomial transformation will have the ability to fit mirroring nonlinearity both below and above zero. Thus, vertical excursion parameters (squares and cubes) and stride durations were tested together and reduced backwards using Wald′s P‐values. Interactions were not tested. Significance was set at P‐value ≤0.05. Residual plots were scrutinised for heteroscedasticity versus the outcome as well as for normality in quantile–quantile plots.

## Results

### General results

Forelimb lameness induction was successful in all 10 horses, as deemed by the HDmin selection criterion (absolute HDmin ≥6 mm). In three horses, left hindlimb induction was not successful (absolute PDmin <3 mm). After induction of hindlimb lameness, six horses showed a compensatory head movement asymmetry (absolute HDmin ≥6 mm) during induction of both left and right hindlimb lameness. Head movement asymmetry (absolute HDmin >6 mm) was also observed in three horses during right hindlimb induction, and in one horse during left hindlimb induction. The mean speed of the trials varied from 3.8 to 4.0 m/s (mean 3.9 m/s).

**Figure 2:**
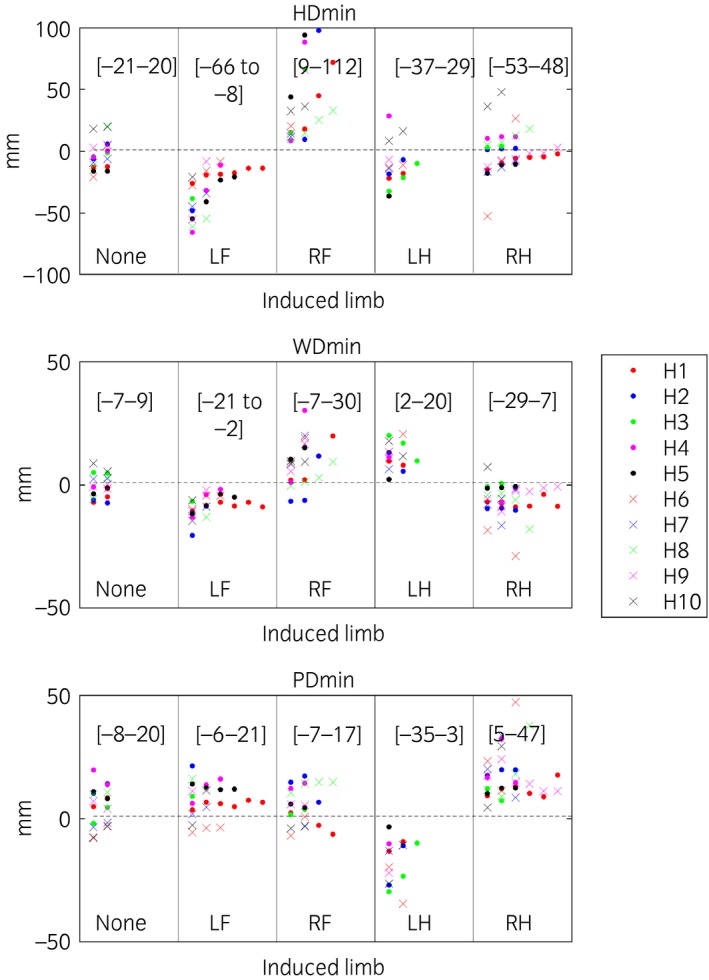
Descriptive plots of mean values per trial for difference between the two position minima per stride of the head (HDmin), withers (WDmin) and pelvis (PDmin). The highest and lowest trial values for each induction are presented within brackets. Lameness was induced in 10 horses (H1–H10), with 1–7 inductions included per limb, where ‘None’ denotes trials before induction and left fore (LF), right fore (RF), left hind (LH) and right hind (RH) denotes the limb induced. A few outliers are hidden as seen from the values of the [ranges] for each parameter and induction.

**Figure 3:**
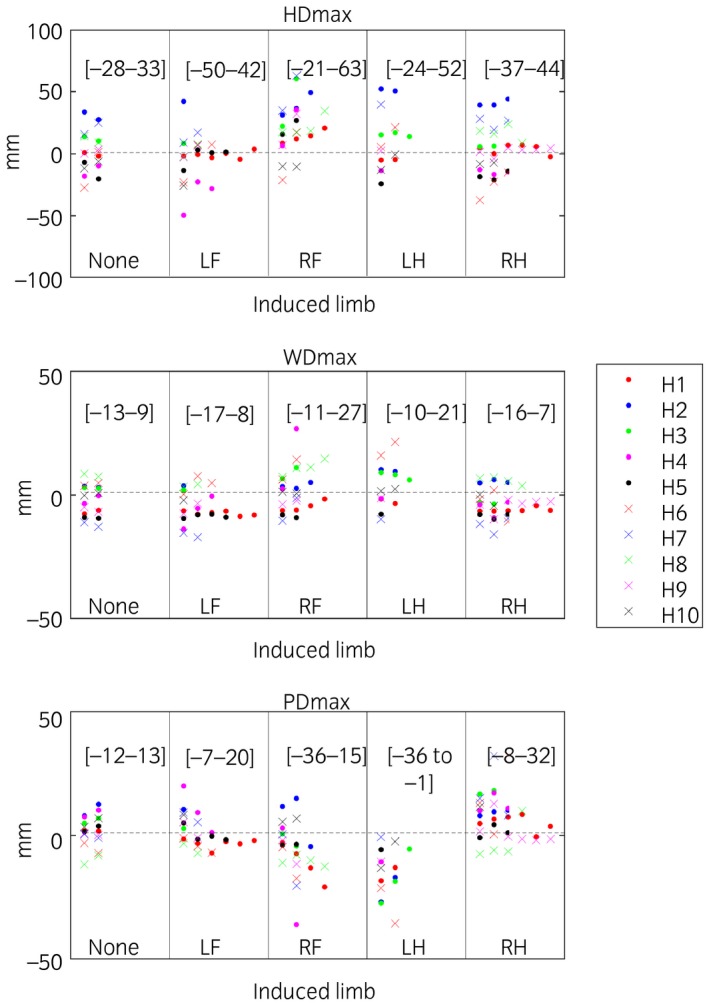
Descriptive plots of mean values per trial for difference between the two position maxima per stride of the head (HDmax), withers (WDmax) and pelvis (PDmax). The highest and lowest trial values for each induction are presented within brackets. Lameness was induced in 10 horses (H1–H10), with 1–7 inductions included per limb, where ‘None’ denotes trials before induction and left fore (LF), right fore (RF), left hind (LH) and right hind (RH) denotes the limb induced. A few outliers are hidden as seen from the values of the [ranges] for each parameter and induction.

Trial mean values for head, withers and pelvic excursion variables (HDmin, HDmax, HDup, WDmin, WDmax, WDup, PDmin, PDmax, PDup) are presented per induced limb, including baseline (before induction of lameness measurements) (Figs 2). For forelimb and hindlimb lameness, head and pelvic variables were plotted, respectively, against withers variables per stride (Fig 3). In these plots, the types of variable (min, max and range‐up differences) were kept separate and plotted against each other. The plots show positive slopes for forelimb inductions and negative for hindlimb inductions.

### Statistical models

Forelimb and hindlimb analysis was performed on data from 1183 and 1182 strides, respectively. The data subset selected for hindlimb lameness with compensatory head movement asymmetry contained 482 strides. Outcomes were best untransformed after normal probability plotting. Stride duration did not confound the estimate of the association between symmetry variables and was hence omitted (Supplementary Item 1).

Model results are presented in Table 1 and associations are shown as lines in Figure 5. In general, the magnitudes of the estimates for PD parameters in the hindlimb analyses were 0.35–0.55 mm and negative. For example, for each mm increase in PDmin in response to hindlimb induction, WDmin increased by 0.55 mm but towards the contralateral side. Conversely, the magnitudes of the estimates for HD parameters in the forelimb analyses were 0.05–0.10 and positive. For example, for each mm increase in HDmin, WDmin increased by 0.05 mm. The positive value indicates an increase in withers movement asymmetry in agreement with the direction of the head movement asymmetry. The fit of the models after residual inspection was considered adequate.

**Table 1:** Model output for forelimb lameness dataset (n strides = 1183), for the hindlimb lameness dataset (n strides = 1182) and for the reduced dataset with induced hindlimb lameness and concurrent compensatory head nod (n strides = 482). The forelimb lameness dataset includes all trials with absolute mean values of HDmin ≥6 mm, the hindlimb dataset all trials with absolute PDmin values ≥3 mm. The reduced dataset includes all trials with hindlimb inductions and concurrent compensatory ipsilateral head movement asymmetry with an absolute HDmin value ≥6 mm

Analysis	Dependent variable	Independent variable	Estimate	SE	P‐value
Forelimb lameness	WDmin	Intercept	−0.09	1.258	0.9
		HDmin	0.05	0.008	<0.0001
	WDmax	Intercept	−1.42	2.272	0.53
		HDmax	0.10	0.008	<0.0001
	WDup	Intercept	−1.26	2.533	0.6
		HDup	0.07	0.009	<0.0001
Hindlimb lameness	WDmin	Intercept	2.98	0.967	0.002
		PDmin	−0.55	0.013	<0.0001
	WDmax	Intercept	−0.69	1.671	0.7
		PDmax	−0.35	0.014	<0.0001
	WDup	Intercept	1.90	1.796	0.3
		PDup	−0.47	0.012	<0.0001
Hindlimb lameness with compensatory head nod	WDmin	Intercept	2.76	1.159	0.02
		PDmin	−0.52	0.019	<0.0001
	WDmax	Intercept	−1.52	2.090	0.5
		PDmax	−0.42	0.024	<0.0001
	WDup	Intercept	1.21	2.735	0.7
		PDup	−0.47	0.019	<0.0001

## Discussion

**Figure 4:**
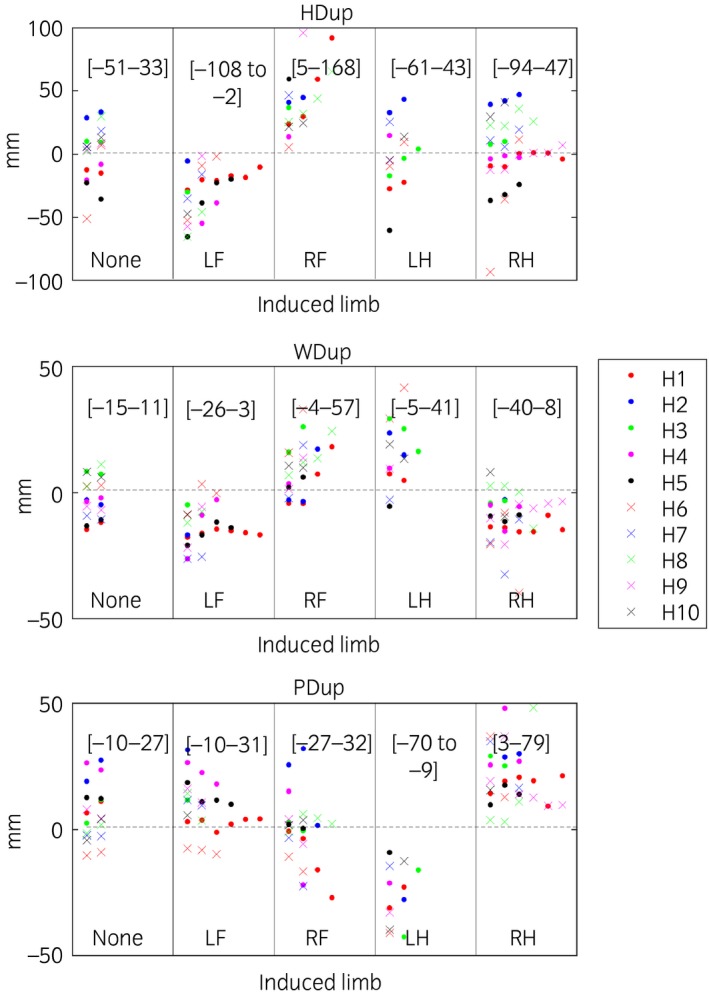
Descriptive plots of mean values per trial for difference between the two upward ranges per stride of the head (HDup), withers (WDup) and pelvis (PDup). The highest and lowest trial values for each induction are presented within brackets. Lameness was induced in 10 horses (H1–H10), with 1–7 inductions included per limb, where ‘None’ denotes trials before induction and left fore (LF), right fore (RF), left hind (LH) and right hind (RH) denotes the limb induced. A few outliers are hidden as seen from the values of the [ranges] for each parameter and induction.

Our data support our hypothesis that head and withers show synchronised asymmetries (indicating the same limb) in horses with forelimb lameness but show movement asymmetries of opposite directions (indicating contra lateral limbs) for hindlimb lameness. These findings are in accordance with Buchner *et al*. 5, who demonstrated that horses with induced forelimb lameness showed synchronous head and withers movement asymmetries, notably reaching a less descended position during the lame forelimb stance (Fig 6). During primary hindlimb lameness, when the lame limb is weight bearing, the trunk is lowered less and consequently maintains a higher position during midstance resulting in changes in both PDmin and WDmin during the lame diagonal stance 5. The head goes down more during the diagonal stance that includes the lame hindlimb, to shift weight to the forehand (the compensatory motion). However, the withers will go down less. This is because the higher position during lame stance in the hindquarter translates diagonally to a higher position of the withers at the contralateral side (the diagonal forelimb) (Fig 4). Hindlimb lameness leads to movement asymmetries of the withers and the head being contralateral. This is different when compared to a primary forelimb lameness. Buchner *et al*. 5 also concluded that withers symmetry was less sensitive for detection of very mild lameness. Our results are in accordance with their conclusion, with the head showing a more exacerbated movement asymmetry compared with withers movement asymmetry. An estimate of 0.05 mm was found for the relationship between HDmin and WDmin (Table 1). However, from the current data, it cannot be concluded whether withers movement symmetry is affected by very mild clinical forelimb lameness.

**Figure 5:**
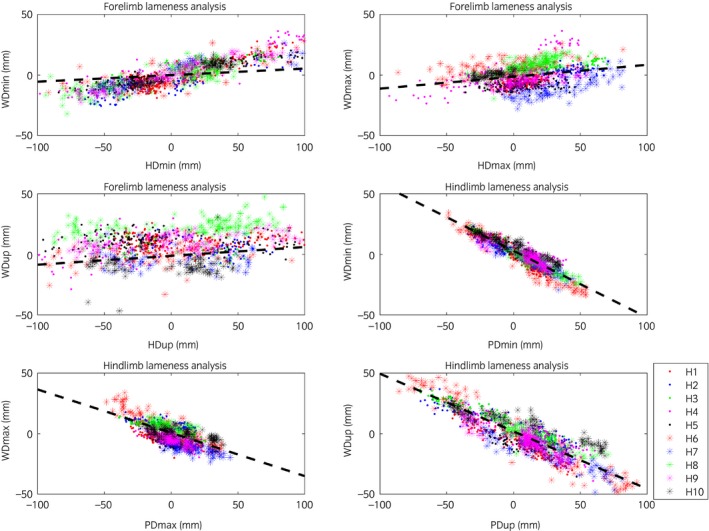
Movement symmetry parameters for stride‐level data for the vertical displacement of the head or pelvis (x‐axis) plotted against vertical displacement of the withers(y‐axis) colour coded by horse (see legend). The lines include the modelled associations from the forelimb and hindlimb lameness models in Table 1. All strides from eachhorse are presented. Some outliers outside the axis limits, which were kept consistent across all panels, are hidden.

**Figure 6:**
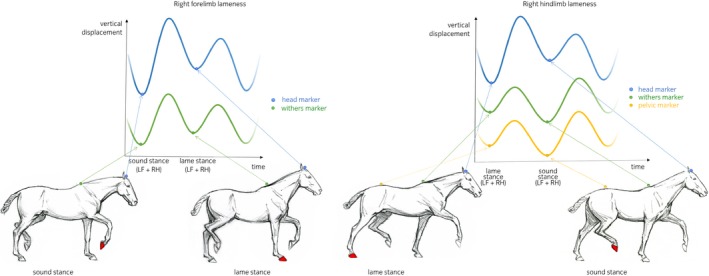
*Primary right forelimb lameness*. The vertical displacement curves of head and withers markers are displayed during one stride in the graph. During the sound diagonal stance (left fore [LF] – right hind [RH] diagonal limb pair), the horse reaches a lower vertical position for both, head and withers compared with the position during the lame diagonal stance where the lame right fore (RF) and left hind (LH) are weight bearing. Primary right hindlimb lameness. The vertical displacement curves of head, withers and pelvic markers are displayed during one stride in the graph. During stance phase of the lame diagonal (right hind [RH] – left fore [LF]), the head exhibits a lower vertical position during lame diagonal stance compared with sound diagonal stance. However, the withers and pelvic markers exhibit a higher vertical position during lame diagonal stance compared with the sound diagonal stance (left hind [LH] – right fore [RF]).

Several studies have shown that horses with clinical or induced primary hindlimb lameness can show a compensatory head movement asymmetry, where the head reaches the lowest position during the diagonal stance phase of the lame hindlimb 8, 9, 11, 18, 19. This head movement is helping the horse decrease the load on the lame hindlimb during stance by transferring weight forward, nodding down more as the lame diagonal is in stance 20, mimicking an ipsilateral forelimb lameness when looking at the head. In the current study, in 8 of 21 trials of induced hindlimb lameness with a compensatory head nod, the mean head movement asymmetry was more substantial compared with the primary pelvic movement asymmetry. This was also seen in the study by Rhodin *et al*. 11 and may be explained by the larger range of motion of the head compared with the pelvis. The prominent compensatory head movement asymmetry, as well as the difficulty of detecting pelvis movement asymmetry, may in more extreme cases result in diagnostic analgesia being performed erroneously on a nonlame forelimb instead of the primary lame hindlimb. False positive interpretations of these blocks due to expectation bias 4, thus resulting in incorrect diagnosis, can have adverse consequences for a horse. There is always a (low but non‐negligible) risk of iatrogenic infections of synovial structures after diagnostic intra‐articular analgesia. Therefore, these procedures may be executed only when deemed necessary. The prevalence of improper localisation of the pathological origin of lameness in the clinical situation is unknown, but it may be substantial. In a study on inter‐observer agreement for veterinarians evaluating lameness during lungeing, the majority of the participants identified a compensatory head movement asymmetry as a sign of primary forelimb lameness in two out of five videos of horses with induced hindlimb lameness on the lunge 12.

In the current study, horses with induced hindlimb lameness and concurrent compensatory head movement asymmetry showed contralateral head and withers movement asymmetries. Therefore, movement symmetry of the withers can be used to discriminate a head nod associated with true forelimb lameness from compensatory head movement asymmetry caused by primary hindlimb lameness.

Kinetic measurements in horses with hindlimb lameness, while having shown an impulse shift to the contralateral forelimb within the lame diagonal, have not shown a decreased loading of the ipsilateral forelimb 20. This would be expected from the kinematic measurements of the head. This may be related to the fact that the forward load redistribution within the diagonal including the lame hindlimb (produced by the accentuated head nod), does not exceed the ‘between diagonal load redistribution’ where the sound diagonal carries more load. An in‐depth analysis of the combined kinetics and kinematics is warranted to understand the complex adaptions of the movements in lame horses.

The movement of the withers can be difficult to observe during straight line trot when obscured by the head or hindquarter when the observer stands in front or behind the horse. Viewing the horse from the side may facilitate the observation of the withers movement, but this is mostly done during lungeing, and we do not know how lungeing influences movement symmetry of the withers in sound or lame horses. The small magnitude of asymmetries of withers movement (compared with head movement) may also play a role when assessing withers movement ‘by eye’ in the light of limitations of the human visual system in perceiving small movement asymmetries 21. Nevertheless, with increasing use of quantitative lameness assessment methods, the ability to measure the vertical displacement of the withers will likely be of additional value in helping with discriminating between forelimb and hindlimb lameness, at least in some cases.

### Limitations of the study

Horses were investigated on a treadmill with a constant speed and it may not be appropriate to extrapolate the results to over ground locomotion where acceleration, deceleration, and movement on a circle might influence compensatory patterns. There is a need to confirm these results in horses with naturally occurring lameness caused by different orthopaedic pathologies and pain arising from other locations in the limb.

## Conclusions

In the current study, horses with induced hindlimb lameness demonstrating a compensatory head movement asymmetry showed motion asymmetries of head and withers indicating lameness in different forelimbs. This is opposite to the situation where horses with induced forelimb lameness showed synchronised head and withers asymmetries. Therefore, movement symmetry of the withers can be used to discriminate a head nod associated with true forelimb lameness from a compensatory head movement asymmetry caused by primary hindlimb lameness. Quantification of withers symmetry may hence aid in localisation of the primary lameness (fore or hind).

## Authors’ declaration of interests

No competing interests have been declared.

## Ethical animal research

The experimental protocol was approved by the Animal Health and Welfare Commission of the canton of Zurich (permission number 51/2013). Horse owners gave consent for inclusion of their animals.

## Sources of funding

Swedish‐Norwegian Foundation for Equine Research (H1247074) and The Swedish Research Council Formas funded the study (2014‐12003‐28225‐26).

## Authorship

M. Rhodin, T. Pfau, L. Roepstorff, M.A. Weishaupt, M.H. Thomsen, A. Egenvall and E. Hernlund designed and executed the study. M. Rhodin, A. Egenvall, F.M. Serra Bragança, E. Persson‐Sjodin, E. Hernlund, and T. Pfau analyzed the data, interpreted the results and prepared the manuscript and all authors revised the manuscript critically and gave the final approval of the manuscript.

## Supporting information


**Supplementary Item 1.** Model output evaluating asymmetry parameters and stride duration.

## Manufacturers' addresses

1 Gedore Group, Remscheid, Germany.

2 Kagra AG, Fahrwangen, Switzerland.

3 Qualisys AB, Motion Capture Systems, Göteborg, Sweden.

4 MathWorks, Natick, Massachusetts, USA.

5 R‐Studio, Boston, Massachusetts, USA.

## References

[1] Hewetson, M. , Christley, R.M. , Hunt, I.D. and Voute, L.C. (2006) Investigations of the reliability of observational gait analysis for the assessment of lameness in horses. Vet. Rec. 158, 852‐857. 16798953 10.1136/vr.158.25.852

[2] Fuller, C.J. , Bladon, B.M. , Driver, A.J. and Barr, A.R.S. (2006) The intra‐ and inter‐assessor reliability of measurement of functional outcome by lameness scoring in horses. Vet. J. 171, 281‐286. 16490710 10.1016/j.tvjl.2004.10.012

[3] Keegan, K.G. , Dent, E.V. , Wilson, D.A. , Janicek, J. , Kramer, J. , Lacarrubba, A. , Walsh, D.M. , Cassells, M.W. , Esther, T.M. , Schiltz, P. , Frees, K.E. , Wilhite, C.L. , Clark, J.M. , Pollitt, C.C. , Shaw, R. and Norris, T. (2010) Repeatability of subjective evaluation of lameness in horses. Equine Vet. J. 42, 92‐97. 20156242 10.2746/042516409X479568

[4] Arkell, M. , Archer, R.M. , Guitian, F.J. and May, S.A. (2006) Evidence of bias affecting the interpretation of the results of local anaesthetic nerve blocks when assessing lameness in horses. Vet. Rec. 159, 346‐348. 16963714 10.1136/vr.159.11.346

[5] Buchner, H.H. , Savelberg, H.H. , Schamhardt, H.C. and Barneveld, A. (1996) Head and trunk movement adaptations in horses with experimentally induced fore‐ or hindlimb lameness. Equine Vet. J. 28, 71‐76. 8565958 10.1111/j.2042-3306.1996.tb01592.x

[6] Kelmer, G. , Keegen, K.G. , Kramer, J. , Wilson, D.A. , Pai, F.P. and Singh, P. (2005) Computer‐assisted kinematic evaluation of induced compensatory movements resembling lameness in horses trotting on a treadmill. Am. J. Vet. Res. 66, 646‐655. 15900946 10.2460/ajvr.2005.66.646

[7] Kramer, J. , Keegan, K.G. , Kelmer, G. and Wilson, D.A. (2004) Objective determination of pelvic movement during hind limb lameness and pelvic height differences. Am. J. Vet. Res. 65, 741‐747. 15198212 10.2460/ajvr.2004.65.741

[8] Uhlir, C. , Licka, T. , Kübber, P. , Peham, C. , Scheidl, M. and Girtler, D. (1997) Compensatory movements of horses with a stance phase lameness. Equine Vet. J. 29, 102‐105. 10.1111/j.2042-3306.1997.tb05065.x 9354301

[9] Maliye, S. and Marshall, J.F. (2016) Objective assessment of the compensatory effect of clinical hind limb lameness in horses: 37 cases (2011–2014). J. Am. Vet. Med. Assoc. 249, 940‐944. 27700267 10.2460/javma.249.8.940

[10] Maliye, S. , Voute, L.C. and Marshall, J.F. (2015) Naturally‐occurring forelimb lameness in the horse results in significant compensatory load redistribution during trotting. Vet. J. 204, 208‐213. 25862395 10.1016/j.tvjl.2015.03.005

[11] Rhodin, M. , Pfau, T. , Roepstorff, L. and Egenvall, A. (2013) Effect of lungeing on head and pelvic movement asymmetry in horses with induced lameness. Vet. J. 198, 39‐45. 10.1016/j.tvjl.2013.09.031 24140227

[12] Hammarberg, M. , Egenvall, A. , Pfau, T. and Rhodin, M. (2016) Rater agreement of visual lameness assessment in horses during lungeing. Equine Vet. J. 48, 78‐82. 25399722 10.1111/evj.12385 PMC4964936

[13] Peloso, J.G. , Stick, J.A. , Soutas‐Little, R.W. , Caron, J.C. , DeCamp, C.E. and Leach, D.H. (1993) Computer‐assisted three‐dimensional gait analysis of amphotericin‐induced carpal lameness in horses. Am. J. Vet. Res. 54, 11535‐11543. 8239146

[14] Kübber, P. , Kastner, J. , Girtler, D. and Knezevic, P.F. (1994) Erkenntnisse über den Einfluß der tiefen Palmarnervanästhesie auf das Gangbild des lahmheitsfreien Pferdes mit Hilfe einer kinematischen Meßmethode. Pferdeheilkunde 10, 11‐21.

[15] Pfau, T. , Noordwijk, K. , Sepulveda Caviedes, M.F. , Persson‐Sjodin, E. , Barstow, A. , Forbes, B. and Rhodin, M. (2017) Head, withers and pelvic movement asymmetry and their relative timing in trot in racing Thoroughbreds in training. Equine Vet. J. 50, 117‐124. 28548349 10.1111/evj.12705 PMC5724686

[16] Merkens, H.W. and Schamhardt, H.C. (1988) Evaluation of equine locomotion during different degrees of experimentally induced lameness. I: Lameness model and quantification of ground reaction force patterns of the limbs. Equine Vet. J. 20, *Suppl* **6**, 99‐106. 9079070 10.1111/j.2042-3306.1988.tb04655.x

[17] Keegan, K.G. , Kramer, J. , Yonezawa, Y. , Maki, H. , Frank Pai, P. , Dent, E.V. , Kellerman, T.E. , Wilson, D.A. and Reed, S.K. (2011) Assessment of repeatability of a wireless, inertial sensor‐based lameness evaluation system for horses. Am. J. Vet. Res. 72, 1156‐1163. 21879972 10.2460/ajvr.72.9.1156

[18] May, S.A. and Wyn‐Jones, G. (1987) Identification of hindleg lameness. Equine Vet. J. 19, 185‐188. 3608952 10.1111/j.2042-3306.1987.tb01371.x

[19] Marshall, J.F. , Lund, D.G. and Voute, L.C. (2012) Use of a wireless, inertial sensor‐based system to objectively evaluate flexion tests in the horse. Equine Vet. J. 44, 8‐11. 23447870 10.1111/j.2042-3306.2012.00611.x

[20] Weishaupt, M.A. , Wiestner, T. , Hogg, H.P. , Jordan, P. and Auer, J.A. (2004) Compensatory load redistribution of horses with induced weight bearing hindlimb lameness trotting on a treadmill. Vet. J. 36, 727‐733. 10.2746/0425164044848244 15656505

[21] Parkes, R.S.V. , Weller, R. , Groth, A.M. , May, S. and Pfau, T. (2009) Evidence of the development of “domain‐restricted” expertise in the recognition of asymmetric motion characteristics of hindlimb lameness in the horse. Equine Vet. J. 41, 112‐117. 19418737 10.2746/042516408x343000

