# Supplementary material for: Vertical movement symmetry of the withers in horses with induced forelimb and hindlimb lameness at trot
Source: Equine Vet J. 2018 May 17;50(6):818–24. doi: 10.1111/evj.12844 (PMC6175082; doi:10.1111/evj.12844)
Supplement: Supplementary file 1 — Supplementary Item 1. Model output evaluating asymmetry parameters and stride duration. [file EVJ-50-818-s001.pdf]

Equine Veterinary Journal

Supplementary Item 1: Model output (number of strides = 1183) in models where asymmetry parameters and stride duration are evaluated for their statistical contribution to the models. The dataset includes all trials with absolute mean values for HDmin ≥6 mm. (For other parameters and models stride duration was not significant.)

Analysis	Dependent variable	Independent variable	Estimate	SE	P-value
Forelimb lameness	WDmax	Intercept	21.15	10.13	0.04
		HDmax	0.10	0.01	<0.0001
		Stride duration (s)	-31.77	13.82	0.02
	WDup	Intercept	34.95	18.53	0.06
		HDup	0.07	0.01	<0.0001
		Stride duration (s)	-50.76	25.74	0.05
